# Fusing the Porcelain

**Published:** 1904-07

**Authors:** J. E. Hancock

**Affiliations:** Chicago.


### FUSING THE PORCELAIN.

By J. E. Hancock, D. D. S., Chicago.

(Written for *The American Journal of Dental Science.*)

In my experience there is nothing which gave me so much trouble and kept me so constantly in "hot water" as learning to properly fuse porcelain, and I do not believe that my experience has been so widely at variance with others. It matters not how carefully or artistically the cavity may be prepared or how accurately the matrix may be burnished or swaged, or how perfectly the shades may be selected and blended, the work all goes for naught and the porcelain becomes a useless mass unless it is properly baked. The usual and disastrous mistake made by beginners (and sometimes by so-called experts) is the *"over fuse."* Better by far have the piece baked just a trifle too little than a trifle too much. In fact, in my own work I seldom carry my heat to the point of complete vitrification, preferirng a "high" biscuit that I may preserve the shades, and relying upon Brewster's "XX" enamel, which is very much lower fusing (and, by the way, like cement it covers a multitude of sins) to give the complete glaze without destroying in any degree the underlying shades of the "high" fusing body and at the same time preserving that translucent effect which we should all try to attain in our inlay work. If an inlay should be *over* fused the piece becomes porous and brittle, the edge strength and shades destroyed and what perhaps would otherwise have been a perfect piece of work becomes nothing more than glass and consequently useless, and now we are compelled to begin all over again and are puzzled to know what the trouble is. The shade guide which accompanies each porcelain outfit is usually correctly fused, and if the student of porcelain will experiment until he can duplicate these the *bake* is pretty sure to be correct. A good and safe rule for the beginner to follow until he becomes sufficiently expert by experience to fuse by the "eye," is what is usually designated as the gold *test.* In other words place upon the fire-clay slab alongside of the piece to be baked a small pellet of pure gold, turn on the heat and on the instant that the gold becomes globular take the time and give so many minutes and seconds before the current is switched off, or if using a gasoline oven so many minutes and seconds before the pressure in the tank is reduced. It has been my observation that no two furnaces fuse in exactly the same time and therefore no given rule can be submitted as to time. Each furnace, of whatever make, must be tested as to time of fusing when purchased and the test may be made in the following manner, viz.: Make a small *pot* of the porcelain evaporating the moisture with perfectly clean piece of white blotting paper, place upon a piece of platinum plate and put on a fire-clay slab. Insert in the furnace placing a small piece of pure gold alongside on the fire clay, turn on the heat and gradually increase until the gold becomes globular, "catch" the time and let it run, say, 30 seconds. Reduce heat until you can see whether or not the piece is fused. If not make another small pot of porcelain and proceed in the same manner as before, adding 5 seconds to the time, and so on until fuse is perfect. Make a note of the procedure and the time required and it will be found that that particular body will come from the furnace of about the same density and glaze each time. It is claimed by some that the fusing of porcelain by the "eye" is a detriment to the sight and should be strenuously avoided. Personally, so far I have had no trouble in this direction, but can readily conceive of the injurious effects which might follow the prolonged inspection of such intense heat. To overcome this Dr. Price of Cleveland, Ohio, has recently placed upon the market a very ingenious instrument which controls and regulates the heat to a nicety without the necessity of the operator's looking into the furnace at all. This instrument, or pyrometer as it is designated, can only be used in connection with the electric oven (and besides is quite expensive), therefore it is useless only where the electric current is obtainable. The proper fusing of porcelain can only be acquired by diligent experimentation and patient observation, and each step in the procedure should be carefully noted and remembered. Dr. Hart J. Goslee (to whom the dental profession is indebted for a large fund of information on these subjects and whose genius I recognize and have the greatest respect for) depreciates the gold test and claims that fusing by the *eye* is the best method extant. For him this may be true, but for the beginner I feel quite sure that the gold *test* is the safest because the student has something tangible by which to judge the procedure. In conclusion I would advise deliberation and care, and time will add the other qualifications.

---

### SETTING PORCELAIN INLAYS.

By J. S. Bridges, D. D. S., Chicago.

(Written for *The American Journal of Dental Science.*)

